# Supplementary material for: A fully automated benchmarking suite to compare macromolecular complexes
Source: Nat Methods. 2025 Dec 22;23(2):387–94. doi: 10.1038/s41592-025-02973-z (PMC12904798; doi:10.1038/s41592-025-02973-z)
Supplement: Supplementary file 1 — Supplementary Figs. 1–9 and Supplementary Sections 1–4. [file 41592_2025_2973_MOESM1_ESM.pdf]

nature methods

Supplementary information https://doi.org/10.1038/s41592-025-02973-z

A fully automated benchmarking suite to compare macromolecular complexes

In the format provided by the authors and unedited

Figure S1: QSMap/QSMapR chain mappings for contact based comparisons. **2**
Figure S2: Auxiliary analysis on QSMap/QSMapR chain mapping algorithms. **3**
S1 QSMapR benchmark **4**
Figure S3: Chain mapping by QSMapR and other tools. 6
Figure S4: Runtime comparisons of QSMap/QSMapR and other tools. 7
Figure S5: QSMapR comparison with US-align using residue-by-residue assignments based on residue numbers. 8
Figure S6: Example limitation of AlphaFold-Multimer based chain mappings in hetero-oligomeric complexes. 9
S2 LDDT summary **10**
S3 QS-score summary **11**
S4 Comparison to reference implementations **12**
Figure S7: Comparison of DockQ v2.1.3 and OpenStructure (OST) DockQ scores 12
Figure S8: Comparison of ICS/IPS scores from Prediction Center and OpenStructure (OST) 14
Figure S9: Comparison of GDT_TS scores from Prediction Center and OpenStructure (OST) 15
Supplementary References **16**

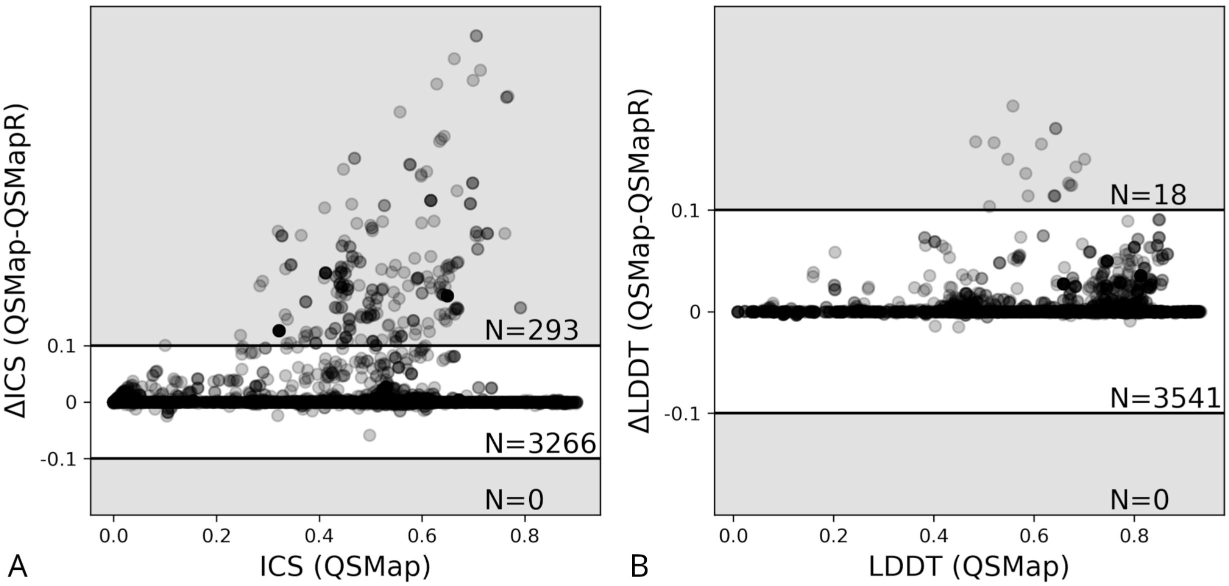

Figure S1: QSMap/QSMapR chain mappings for contact based comparisons.

(A) QSMap gives better chain mappings than QSMapR based on the interface contact based ICS score. (B) A similar trend is observed for LDDT scores, though the improvement is less pronounced, as LDDT operates on both interfacial and intra-chain contacts. The latter is often dominating but is less sensitive to chain mapping differences.

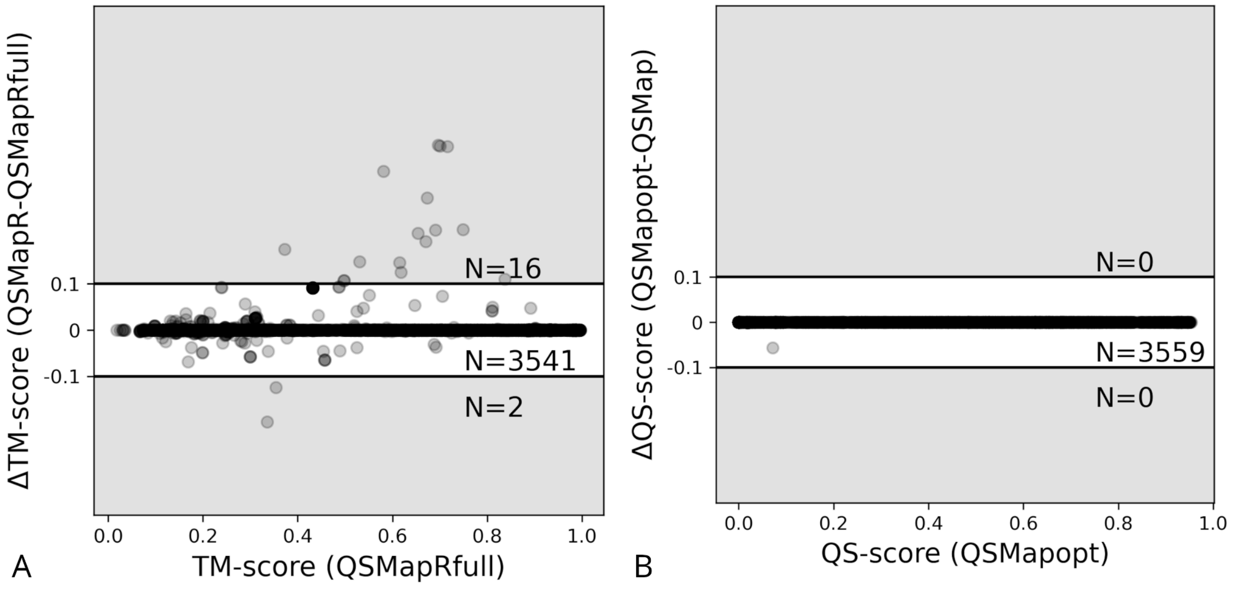

Figure S2: Auxiliary analysis on QSMap/QSMapR chain mapping algorithms.

(A) Comparison of QSMapR with subsampling of n=50 (default) and QSMapRfull which does not include subsampling. (B) To avoid being trapped in local optima in QSMap, we explored a technique described for US-align: after every N chain assignments (N=3), all pairwise swaps among already assigned chains were evaluated for potential score improvement. However, this approach did not enhance chain mappings and was discarded.

S1 QSMapR benchmark

We benchmarked QSMapR on CASP15 data, as described in the main manuscript, comparing it against US-align [1], Foldseek-Multimer [2], and the algorithm described for AlphaFold-Multimer [3] in terms of their ability to establish a chain mapping that optimizes the global superposition-dependent TM-score, as well as runtime performance.

All benchmarks were performed using a single thread of an AMD EPYC 7742 processor. To minimize the impact of filesystem performance, structure files were copied to fast local scratch discs prior to computation.

QSMapR generates chain mappings that are similar to those produced by US-align (Figure S3A), but operates approximately an order of magnitude faster (Figure S4A). In contrast, when compared to Foldseek-Multimer and the method used by AlphaFold-Multimer, QSMapR yields significantly more accurate chain mappings (Figure S3B/C), albeit with longer runtimes than AlphaFoldMultimer (Figure S4B). While Foldseek-Multimer exhibits longer runtimes for smaller problem sizes, its runtime scales more efficiently with increasing size, eventually outperforming QSMapR for larger problems (Figure S4B).

Details of the benchmark are available in the subsequent paragraphs.

Computing TM-score: The `-chainmap` flag in US-align, which was kindly implemented by Chengxin Zhang upon request, allows one to compute an optimal TM-score for a given chain mapping:

```
USalign <mdl> <ref> -mm 1 -ter 0 -chainmap <mapping>
```

where `<mapping>` is the path to a file with each line defining a tab separated model/reference chain assignment. The TM-score is computed in a sequence independent manner, optimizing residue assignments in order to maximize TM-score.

Executing US-align chain mapping: US-align (Version 20241108) was compiled from source using the recommended compilation flags, as available at https://github.com/pylelab/USalign. The following command was used to perform sequence-independent optimization and was executed via Python’s subprocess module:

```
USalign <mdl> <ref> -mm 1 -ter 0
```

Runtime was extracted directly from US-align output. The default command performs a fully sequence-independent comparison which is somewhat unfair, as QSMap/QSMapR perform a sequence based residue-by-residue assignments as well as pre-grouping that reduces chain mapping complexity for the case of hetero-oligomers. To partially address this discrepancy, we repeated the analysis using the following US-align command:

USalign <mdl> <ref> -TMscore 6

This enforces residue-by-residue assignment (byresi) between mapped chain pairs based on residue numbers. This approach is valid for our benchmark dataset, as residue numbers in the models are numbered according to the underlying reference sequences. However, since this mode still lacks sequence-based pre-grouping, a disadvantage for US-align remains.

Executing Foldseek-Multimer chain mapping: Foldseek (Version b4f14464bd9e3fb1921779921b985790955acd2e) was downloaded as a pre-compiled tarball using the command provided at https://github.com/steineggerlab/foldseek:

```
wget https://mmseqs.com/foldseek/foldseek-linux-avx2.tar.gz
```

Foldseek applies efficient prefilters to minimize the number of actual chain mappings that need to be computed, thus allowing efficient searches of large databases. However, to mimic its application in a benchmarking scenario, we explicitly compute chain mappings on model/reference pairs using the following command executed via Python's subprocess module:

```
Foldseek easy-multimersearch <mdl> <ref> <res> <tmp>
```

where `<res>` is the prefix for the generated results files and `<tmp>` is a temporary directory. Runtime was computed based on timestamps before and after the subprocess call. As for US-align, Foldseek-Multimer also does not perform a sequence-based pre-grouping which might deliver suboptimal results for a benchmarking scenario.

Executing Alphafold-Multimer: We implemented the algorithm described in section 7.3 of the Alphafold-Multimer manuscript [3] in OpenStructure. To summarize (largely follows the description in the Alphafold-Multimer manuscript):

One anchor chain in the ground truth (reference) is selected. If the reference has stoichiometry A3B2, an arbitrary chain in B is selected as it has smaller ambiguity. In a tie, for example A2B2, the longest chain in A, B is selected.

Given a model chain with the same sequence as the reference anchor chain, a CA-RMSD (C3' for nucleotides) based superposition is performed. Model chains are then greedily assigned to reference chains of equivalent sequence by minimum distance of their geometric centers. This procedure is repeated starting from every model chain with the same sequence as the reference anchor chain. The assignment leading to minimal RMSD of the geometric centers is returned.

A modified version of this algorithm supports different stoichiometries between the reference and the model. During anchor selection, OpenStructure ensures that at least one model chain with the same sequence can be mapped to the selected reference chain. If no valid mapping

exists for the smallest stoichiometric group in the reference, the algorithm proceeds to the next smallest group, and so on.

We invoked this chain-mapping procedure directly via the OpenStructure Python module, measuring runtime using timestamps before and after execution. However, we did not integrate the algorithm as an option into OpenStructure's scoring actions as it does not produce optimal chain mappings for global superposition-based metrics such as TM-score. One example limitation is the reliance on a single anchor chain in the reference that can lead to suboptimal results if none of the model chains accurately represents that anchor (Figure S6A/B).

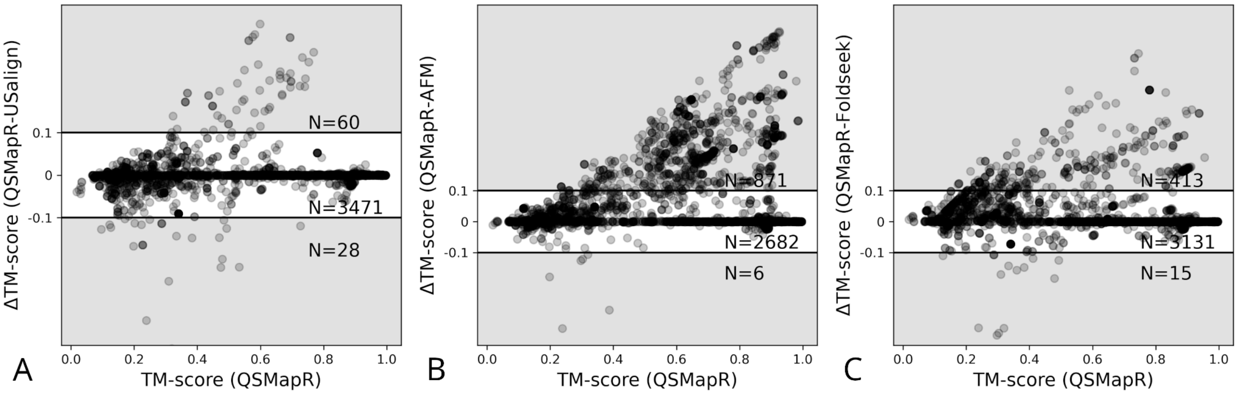

Figure S3: Chain mapping by QSMapR and other tools.

Shown are results for (A) US-align, (B) the AlphaFold-Multimer chain mapping algorithm implemented in OpenStructure, and (C) Foldseek-Multimer. Algorithms were executed as described above.

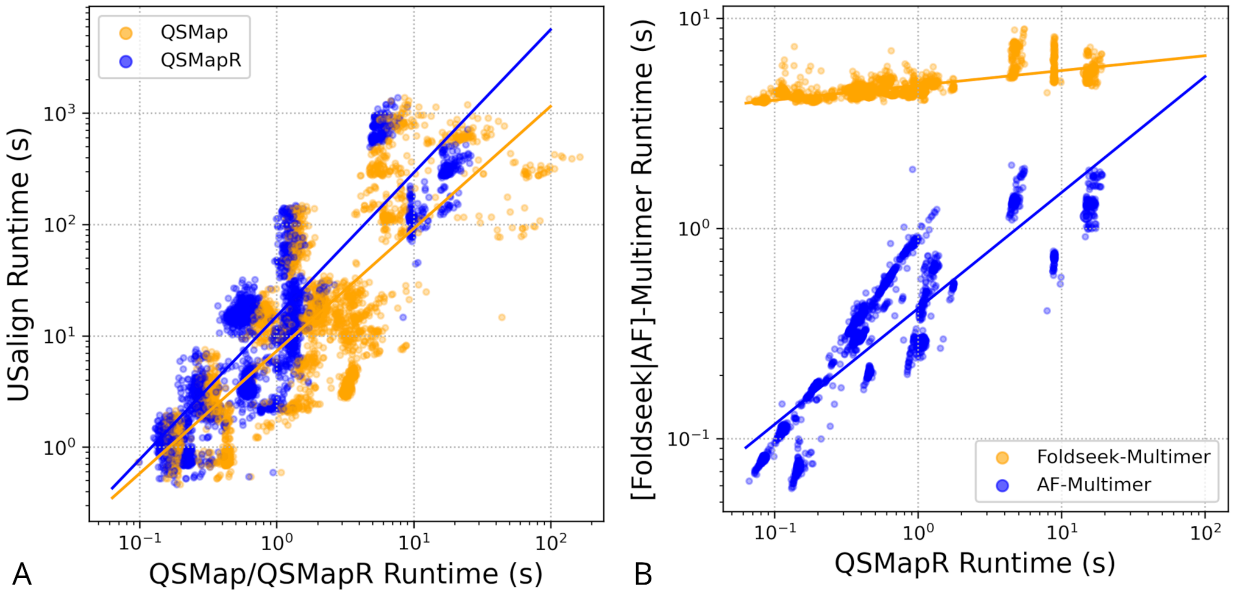

Figure S4: Runtime comparisons of QSMap/QSMapR and other tools.

Shown are results for (A) QSMap/QSMapR vs US-align and (B) QSMapR vs AlphaFold-Multimer chain mapping algorithm implemented in OpenStructure and Foldseek-Multimer. Timings were collected as described above.

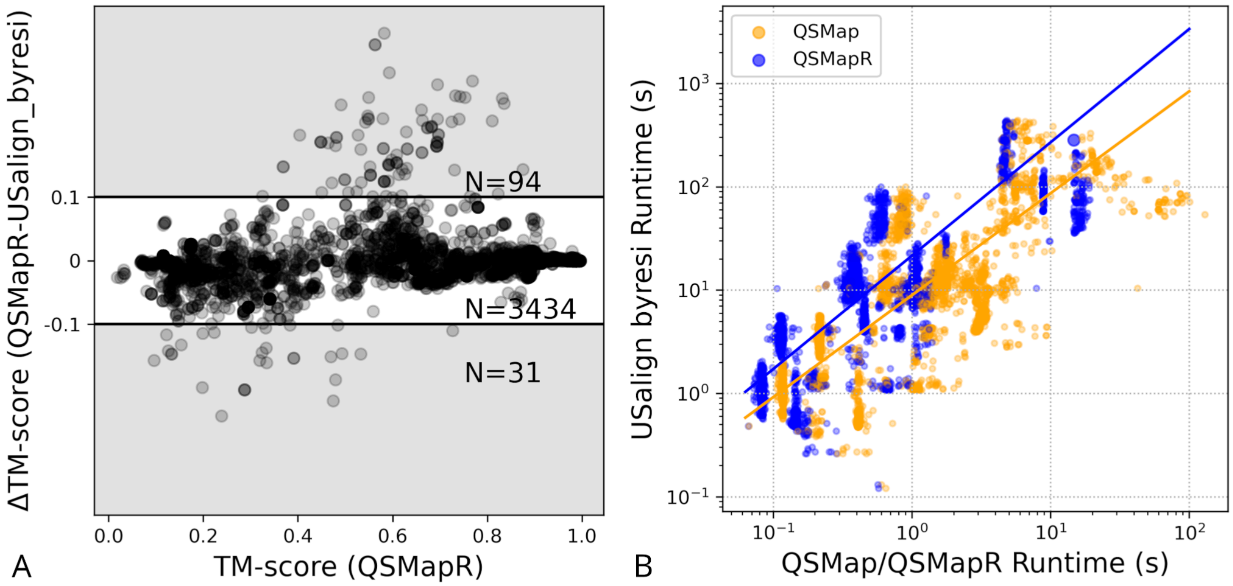

Figure S5: QSMapR comparison with US-align using residue-by-residue assignments based on residue numbers.

(A) Reproduction of Figure S3A and (B) reproduction of Figure S4A when using US-align with enforced residue-by-residue assignment (byresi) between mapped chain pairs based on residue numbers.

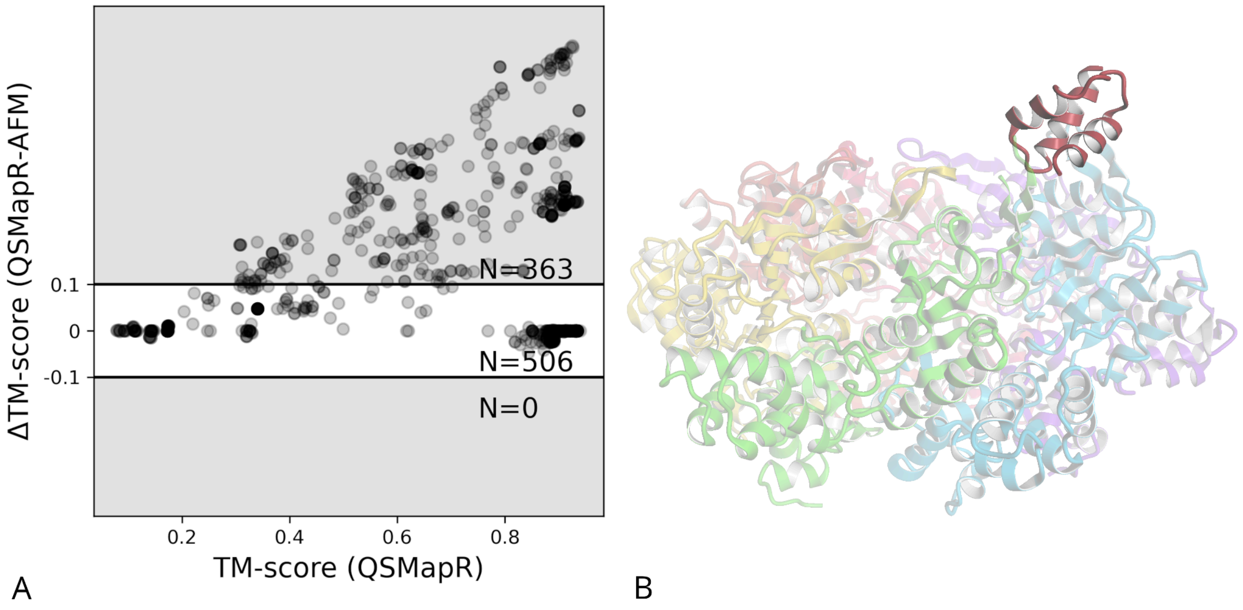

Figure S6: Example limitation of AlphaFold-Multimer based chain mappings in hetero-oligomeric complexes.

(A) Reproduction of Figure S3B using a subset of models from CASP15 hetero-oligomeric targets H1171, H1172 and H1135 with stoichiometries A6B1, A6B2 and A9B3. In all cases, the smaller group, from which the AlphaFold-Multimer chain mapping algorithm selects the reference anchor, represents small protein domains. (B) Reference structure of CASP 15 hetero-oligomeric target H1171 (PDB ID: 7PBL). The red-colored chain, selected as the reference anchor, is frequently misoriented in predicted models, leading to suboptimal chain mappings.

S2 LDDT summary

For each interatomic distance ≤15 Å in the reference structure, a comparison is made with its corresponding counterpart in the model. LDDT calculates the fraction of distance differences that fall below a threshold *d.* Distances involving atoms that are not present in the model are considered unfulfilled, i.e. above the threshold. The finally reported values are arithmetic averages of four fractions calculated using thresholds [0.5, 1.0, 2.0, 4.0].

As a preprocessing step, LDDT evaluates stereochemistry, checking the model for significant stereochemical irregularities such as uncommon bond lengths/angles and clashes. Ideal bond lengths and angles, along with their standard deviations, are hardcoded for proteinogenic amino acids according to Engh and Huber [4]. Deviations exceeding 12 standard deviations are considered serious violations. Additionally, interatomic distances between pairs of non-bonded atoms in the model are considered clashing if the distance between them is smaller than the sum of their corresponding atomic van der Waals radii (Allen, 2002), within a predefined tolerance threshold (by default 1.5 Å). LDDT is penalized by removing all sidechain atoms if any of the sidechain atoms is involved by such irregularities or by removing all atoms of the entire residue if the backbone is involved (backbone definition for amino acids: N, CA, C, O, all other atoms are considered sidechain atoms). The latter results in a per-residue LDDT of 0.0.

Some residues are symmetric, i.e. allow different mappings between reference and model that are chemically equivalent. One example are the OD1/OD2 atoms in aspartic acid (ASP). Symmetries in proteinogenic amino acids can additionally be found in glutamic acid (GLU), leucine (LEU), valine (VAL), arginine (ARG), phenylalanine (PHE) and tyrosine (TYR). In LDDT, residues with symmetric atoms are pre-processed by calculating the LDDT score for the symmetric atoms relative to all fixed atoms (i.e., atoms from other residues that are not symmetric). Two computations are performed: LDDT_1 uses the mapping based on the original atom naming and LDDT_2 a swapped mapping (e.g. OD1 and OD2 in ASP are interchanged). The higher scoring mapping is then used in the final LDDT computation.

S3 QS-score summary

The quaternary structure score (QS-score, [5]) quantifies similarities between two protein complexes as a function of shared interface contacts. It is similar to the Q function [6,7] but other than Q and various other metrics assessing individual interfaces, QS-score is not restricted to dimers. It assesses all interfaces at once and is thus suited to compare complexes of arbitrary size, even when they differ in their number of chains. Notably, QS-score is symmetric, QS-score(A,B)=QS-score(B,A), and it penalizes contacts missing in one structure. As a result, maximal QS-scores are achieved only when the stoichiometries of the compared complexes are identical. QS-score requires a one-to-one mapping between chains in the two structures.

In detail: QS-score operates on interface contacts which are defined as two residues from different chains with Cβ distance (Cα in case of Glycine) <= 12Å. A contact is considered shared if it occurs in the reference (*A*) and the model (*B*) structure which allows computing a shared score (*S*) based on distance differences:

$$S(A,B) = \frac{\sum_{shared(A,B)} w(min(d_A, d_B)) \left(1 - \frac{|d_A - d_B|}{12}\right)}{\sum_{shared(A,B)} w(min(d_A, d_B))}$$

With *d* being distance and *w* a weighting function that described the probability that two residues across interfaces interact [7]:

$$w(d) = \begin{cases} 1 & \text{if } d \leq 5 \\ e^{-2\left(\frac{d-5}{4.28}\right)^2} & \text{if } d > 5 \end{cases}$$

QS-score is symmetric and penalizes for non-shared contacts that only occur in one of the structures. In the example of the reference (A), this gives a non-shared score (*NS*):

$$NS(A) = \sum_{non-shared(A)} w(d_A)$$

From which follows the full QS-score:

$$QS - score(A,B) = \frac{S(A,B)}{NS(A) + NS(B)}$$

This work updates the reference implementation of QS-score and adapts it to the needs of the QSMap chain mapping algorithm. In short: computation of QS-score is strictly separated into interface by interface contributions.

S4 Comparison to reference implementations

DockQ/ f_{nat}/ iRMS/ LRMS: OpenStructure aims to be an exact clone of these scores which are designed to assess two-body problems, i.e. dimers. We compiled a testset of 6408 models from 23 dimer targets in the CASP15 assembly prediction category [8] to compare the OpenStructure implementations with DockQ v2.1.3 available from https://github.com/bjornwallner/DockQ. Chain mapping derived from QSMap was set as a command line parameter in DockQ. Results from OpenStructure closely match the ones from DockQ v2.1.3 (Figure S7).

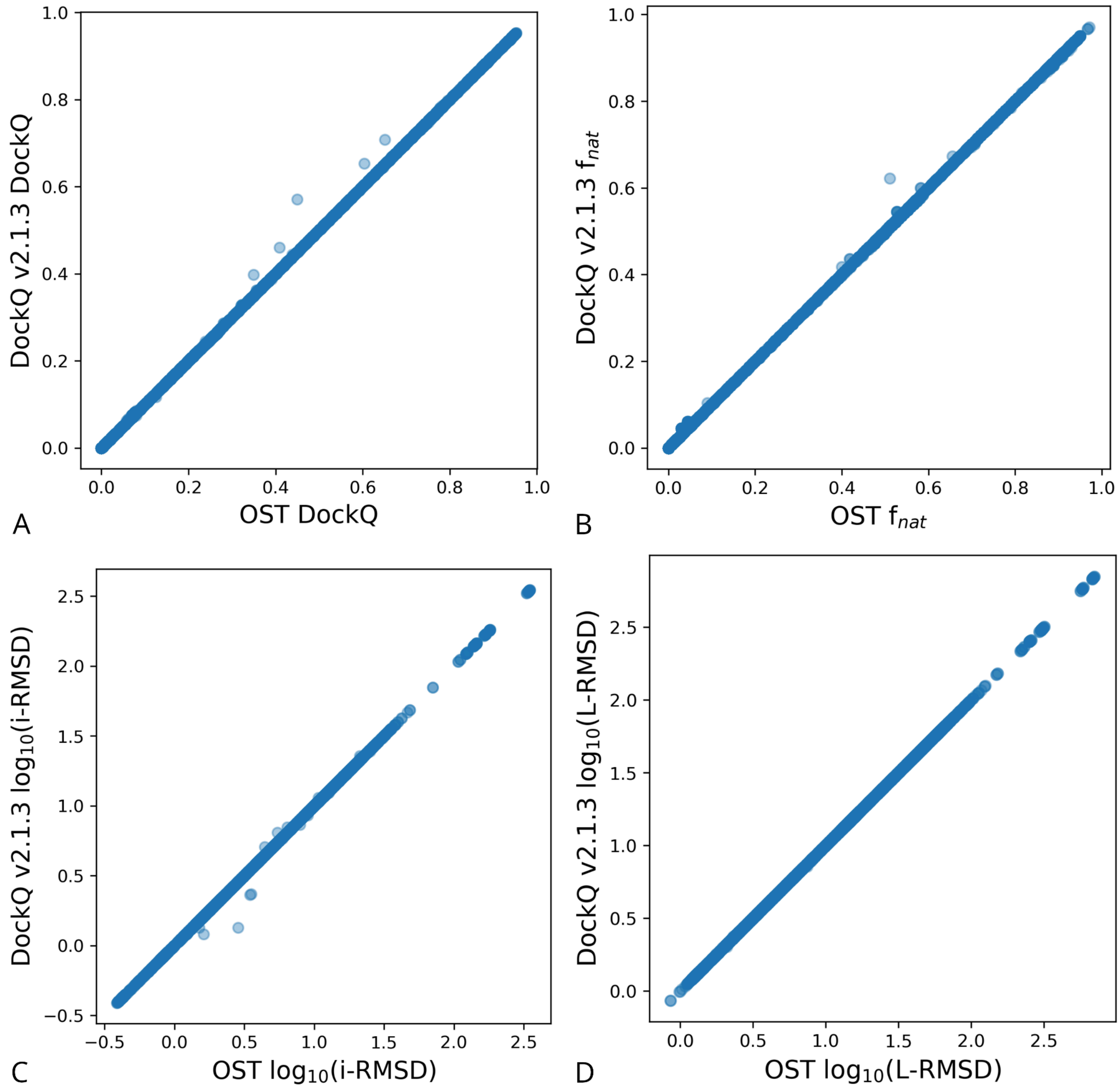

Figure S7: Comparison of DockQ v2.1.3 and OpenStructure (OST) DockQ scores

on 6406 CASP15 dimer structure model/reference pairs. Closely matching values for DockQ (A) and all scores contributing to it: f_{nat} (B), iRMSD (C) and lRMSD (D).

ICS/IPS: We compiled a testset of 11122 models from 41 assembly targets in the CASP15 assembly prediction category [8] to compare the OpenStructure implementations with scores reported by the Prediction Center (https://predictioncenter.org/download_area/CASP15/results/tables/oligo.tar.gz). Results from OpenStructure closely match for dimers. Results for higher order assemblies are qualitatively similar, with discrepancies likely due to differences in chain mapping and score aggregation (Figure S8) with further details available in section “Implementation of external scores in OpenStructure” of the online methods.

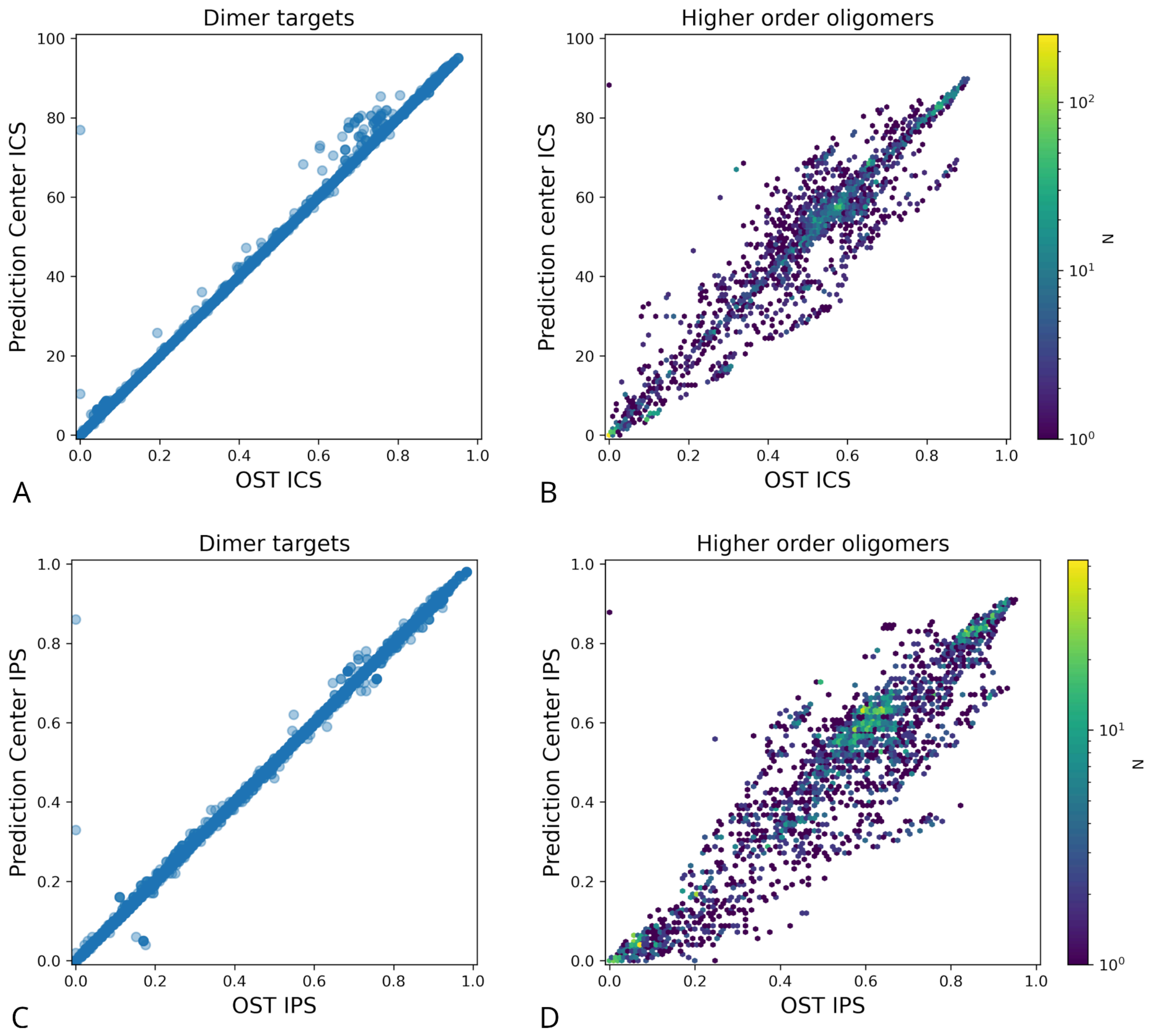

Figure S8: Comparison of ICS/IPS scores from Prediction Center and OpenStructure (OST)

on 6323 CASP15 dimer structure model/reference pairs and 4627 higher order oligomer model/reference pairs. Scores for dimers closely match (A/C) and scores for higher order oligomers are qualitatively similar (B/D).

GDT: Since LGA does not support oligomers, we used all CASP15 tertiary structure models with domain-split reference structures as provided by the CASP15 organizers (20644 data points) to compare GDT_TS from OpenStructure with GDT_TS scores reported by the Prediction Center (https://predictioncenter.org/download_area/CASP15/results/sda/). The scores closely match, with 99.5% of the values falling within 2 points of each other on a scale of 0 to 100. On average (arithmetic average), GDT_TS scores obtained from the Prediction Center were 0.21 points higher than those from OpenStructure, with the largest discrepancies observed in models with lower GDT_TS scores (Figure S9).

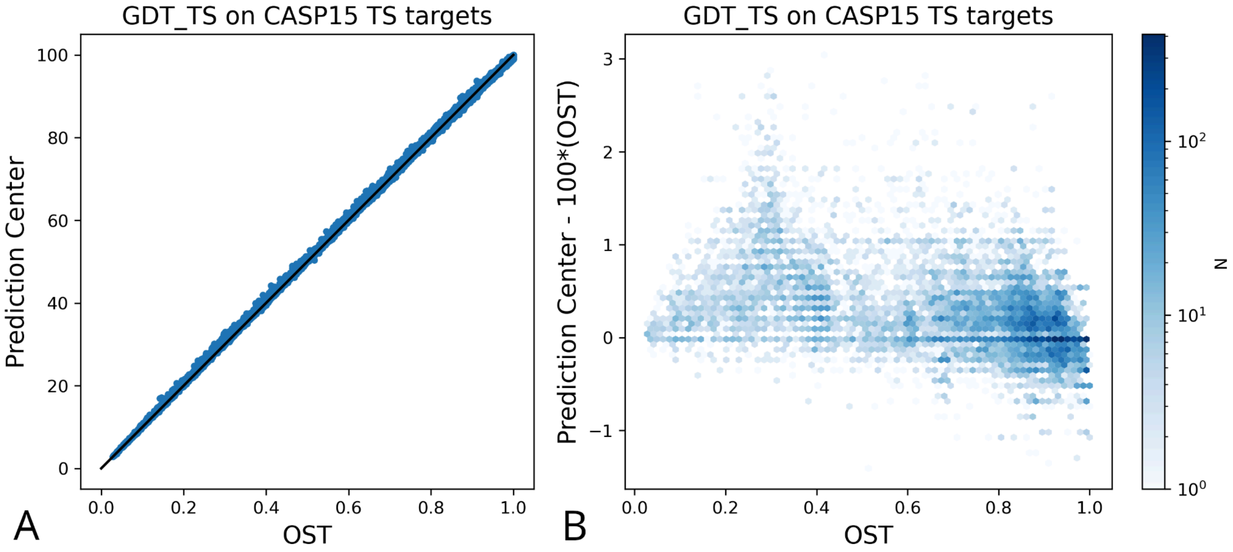

Figure S9: Comparison of GDT_TS scores from Prediction Center and OpenStructure (OST)

on 20644 CASP15 tertiary structure model/reference pairs. (A) The scores closely match, with 99.5% of the values falling within 2 points of each other on a scale of 0 to 100. (B) On average, GDT_TS scores obtained from the Prediction Center were 0.21 points higher than those from OpenStructure, with the largest discrepancies observed in models with lower GDT_TS scores.

Supplementary References

1. Zhang, C., Shine, M., Pyle, A. M. & Zhang, Y. US-align: universal structure alignments of proteins, nucleic acids, and macromolecular complexes. *Nat Methods* **19**, 1109–1115 (2022).
2. Kim, W. *et al.* Rapid and sensitive protein complex alignment with Foldseek-Multimer. *Nat Methods* **22**, 469–472 (2025).
3. Evans, R. *et al.* Protein complex prediction with AlphaFold-Multimer. *bioRxiv* (2021) doi:10.1101/2021.10.04.463034.
4. Engh, R. A. & Huber, R. Accurate bond and angle parameters for X-ray protein structure refinement. *Acta Crystallogr. A* **47**, 392–400 (1991).
5. Bertoni, M., Kiefer, F., Biasini, M., Bordoli, L. & Schwede, T. Modeling protein quaternary structure of homo- and hetero-oligomers beyond binary interactions by homology. *Sci Rep* **7**, 10480 (2017).
6. Xu, Q., Canutescu, A., Obradovic, Z. & Dunbrack, R. L., Jr. ProtBuD: a database of biological unit structures of protein families and superfamilies. *Bioinformatics* **22**, 2876–2882 (2006).
7. Xu, Q. *et al.* Statistical analysis of interface similarity in crystals of homologous proteins. *J Mol Biol* **381**, 487–507 (2008).
8. Ozden, B., Kryshtafovych, A. & Karaca, E. The Impact of AI-Based Modeling on the Accuracy of Protein Assembly Prediction: Insights from CASP15. *bioRxiv* (2023) doi:10.1101/2023.07.10.548341.
